# Editorial: Advances in neuroradiology: emerging diagnostic and therapeutic innovations

**DOI:** 10.3389/fradi.2026.1984316

**Published:** 2026-09-22

**Authors:** Corrado Ini’, Silvia Gigli, Abhishek Mahajan, Lorenzo Aliotta, Emanuele David

**Affiliations:** 1 Radiology I Unit, University Hospital A.O.U. Policlinico “G. Rodolico-San Marco”, Catania, Italy; 2 Department of Diagnostic Imaging, Sandro Pertini Hospital, Rome, Italy; 3 Department of Radiology, The Clatterbridge Cancer Centre, Liverpool, United Kingdom; 4 Radiology I Unit, University Hospital A.O.U. Policlinico “G. Rodolico-San Marco”, Department of Medical, Surgical Sciences and Advanced Technologies “G.F. Ingrassia”, Catania, Italy

**Keywords:** artificial intelligence, cerebral microbleeds, diagnostic imaging, magnetic resonance imaging, neuroradiology, pediatric neuroimaging, quantitative imaging, translational research

Neuroradiology plays a pivotal role within the field of diagnostic imaging, given the significance of the conditions treated and their prevalence, as well as the giant strides made in both diagnostic and interventional procedures, which have contributed to improved clinical outcomes for many neurological disorders.

In an era characterised by an ever-increasing need for objectivity expressed in quantitative and semi-quantitative terms, as well as for artificial intelligence, the diagnostic and therapeutic demands placed on neuroradiology are constantly growing. As imaging hardware, quantitative post-processing, and artificial intelligence continue to advance, the field faces persistent challenges: the limitations of existing imaging modalities, the need to improve accuracy in disease detection, and the ongoing effort to refine treatment approaches in order to develop a tailored approach and enhance patient outcomes. This Research Topic was launched to capture this momentum, inviting contributions on the role of artificial intelligence and machine learning in neuroradiology, innovations in imaging techniques and their clinical applications, cross-disciplinary approaches enhancing diagnostic and therapeutic precision, translational research linking diagnostic imaging with treatment protocols, and the challenges and opportunities involved in implementing new neuroradiological practices. The seven contributions gathered in response to this call illustrate how these themes translate into practice, refining diagnostic accuracy and shaping clinical decision-making across the neurological life span, from the neonatal brain to rare adult pathologies.

A recurring theme across the Topic is the drive to make neuroimaging more quantitative and reproducible. Chandran et al. compared deep learning image reconstruction (DLIR) with conventional iterative reconstruction in non-contrast CT of the head, showing that DLIR simultaneously improved gray-white matter differentiation, signal-to-noise and contrast-to-noise ratios, and lesion conspicuity, while allowing a substantial reduction in radiation dose—an important step toward dose-sparing protocols in a population subjected to repeated CT surveillance. Complementing this technical advance, Tahsini et al. moved quantitative spectroscopy into the diagnostic algorithm for Canavan disease, establishing cutoff values for NAA/Cr and NAA/Cho ratios on proton MR spectroscopy that achieved excellent diagnostic accuracy in a single-center cohort. Both studies exemplify a shift from qualitative pattern recognition toward objective, threshold-based imaging biomarkers.

Cerebral microbleeds emerged as a unifying imaging marker in two contributions addressing very different vascular substrates. Xu et al. reported a patient with CADASIL in whom persistent vessel wall enhancement on high-resolution vessel wall MRI accompanied progression of microbleeds, supporting a role for blood-brain barrier disruption in the pathophysiology of this hereditary cerebral small vessel disease; although derived from a single case and requiring confirmation in larger series, this observation raises the hypothesis that vessel wall imaging could serve as a longitudinal biomarker of disease activity alongside established markers such as white matter hyperintensity burden. Bognár et al., in their mini review, synthesized the heterogeneous literature on cerebral microbleeds—detected using susceptibility-sensitive sequences such as gradient-echo and susceptibility-weighted imaging—as markers of traumatic axonal injury, an entity independently characterized using diffusion imaging techniques such as diffusion tensor imaging. Their synthesis highlights both the promise and the current inconsistencies in using microbleeds as a marker of injury severity after traumatic brain injury. Read together, these two contributions underscore that microbleeds are not a single entity but a context-dependent signal whose interpretation must be anchored to the underlying vascular pathology.

Two contributions addressed the particular challenges of pediatric neuroimaging. Di Stasi et al. provided a practical guide for the non-pediatric neuroradiologist encountering neonates treated with therapeutic hypothermia for hypoxic-ischemic encephalopathy, summarizing optimal imaging timing, protocols, and characteristic findings for a treatment increasingly delivered outside dedicated pediatric centers, guidance that is particularly valuable as therapeutic hypothermia becomes standard of care in general neonatal units without on-site pediatric neuroradiology expertise. Lin et al. described a rare presentation of Sturge-Weber syndrome type III in a 5-year-old child without the cutaneous or ocular stigmata typical of the syndrome, in whom multimodal imaging – CT for calcifications, and MRI with susceptibility-weighted and contrast-enhanced sequences for leptomeningeal vascular malformation—proved decisive for diagnosis despite nonspecific neurological symptoms. Both papers reinforce imaging's central role when clinical presentation alone is insufficient to guide diagnosis in children.

Finally, Karasev et al. presented a diagnostically challenging case of gliosarcoma, a rare and aggressive IDH-wildtype glioblastoma variant, in which T2-weighted heterogeneity of the solid tumor component—reflecting the biphasic glial and mesenchymal composition of the tumor—offered a potential radiological clue to a diagnosis that otherwise closely mimics glioblastoma, solitary metastasis, or anaplastic meningioma on conventional sequences. Even if this observation derives from a single case and needs validation in larger cohorts, imaging features of this tumor may usefully inform preoperative counselling and surgical planning given the differing prognosis and management pathways associated with gliosarcoma relative to its radiological mimics.

Taken together, the articles in this Research Topic reflect the breadth of contemporary neuroradiology: from dose-optimized, AI-assisted CT reconstruction and quantitative spectroscopy, to vascular imaging biomarkers spanning hereditary small vessel disease and trauma, to the diagnostic value of multimodal imaging in rare pediatric and oncologic presentations. Several directions for future work emerge from this Research Topic. Quantitative and AI-based tools, such as those described for CT dose reduction and MRS-based diagnosis, will require multicenter, prospective validation before routine incorporation into diagnostic algorithms, together with harmonization of acquisition protocols across scanners and institutions. Imaging biomarkers such as cerebral microbleeds call for pathology-specific interpretive frameworks rather than a one-size-fits-all approach, and would benefit from standardized definitions and reporting criteria to enable comparison across cohorts and diseases. Rare and atypical presentations—whether a pediatric neurocutaneous syndrome without cutaneous findings or an uncommon glioblastoma variant—continue to depend on the neuroradiologist's pattern recognition and multimodal integration, even as automated tools mature, and case-based literature such as that presented here remains essential for building the collective experience needed to recognize these entities in daily practice. Looking ahead, translating these findings into clinical benefit will depend on closer collaboration between neuroradiologists, clinicians, and data scientists to embed validated quantitative biomarkers into routine reporting workflows, ultimately supporting earlier diagnosis and more personalized therapeutic strategies for patients with neurological disorders.

We thank all authors and reviewers who contributed to this Research Topic and hope that these articles stimulate further translational research bridging diagnostic innovation with improved therapeutic strategies in neuroradiology.

